# Fracture of the Atlas: Separation of a Fragment, and Its Subsequent Extrusion through the Mouth

**Published:** 1913-01

**Authors:** Roswell Park


### Fracture of the Atlas: Separation of a Fragment, and Its Subsequent Extrusion Through the Mouth

### The Unique Case of Dr. James P. White

Reported by ROSWELL PARK

There came into my possession some twenty years ago, perhaps longer, the subjoined statements regarding the nature of a very unusual accident, with still rarer sequels, which befell Dr. James P. White, one of the founders of the Buffalo General Hospital, during the year 1837. In December of that year something happened to the stage-coach in which he was riding, near Batavia, and he was violently thrown, and in such a way as to seriously injure his head and neck. I have not been able to learn any of the details either of the event or of his subsequent symptoms. But this case is not only of extreme interest from the surgeon's viewpoint but peculiarily so to us because of past associations. There is no reason why these documents may not now be reprinted. I regret the impossibility of adding clinical details. However no one now or recently living can furnish them. Nevertheless their authenticity is such as to make it incontestible that the atlas may be fractured and a segment be discharged later through the pharynx, and that the victim of the accident may live for many years without suffering material inconvenience. Those who still remember Dr. White can testify to his unimpaired usefulness and to unceasing activity of his daily life.

The following certificate was published in the Philadelphia Medical News of November 27th, 1886. The original, with Joseph Pancoast's own signature, witnessed by Morris Stroud French, M. D., of Philadelphia, is in my possession. If I recall correctly it was put in my hands by the late Dr. J. B. Andrews. How it fell into his I do not remember.

"An Interesting and Remarkable Accident."

"There came, accidentally, into our possession, a short time age, a memorandum, signed by the late Joseph Pancoast, relating to a remarkable injury received by the late James P. White, the well-known gynecologist, of Buffalo. The note was as follows:

"A front segment of the atlas vertebra, a little more than an inch on the superior margin, a little less below, with the facette which received the odontoid process. It was in the possession of Professor Granville S. Pattison, to whom it was loaned by Dr. White, to show Professors Joseph Pancoast and McClellan. It is probable that the transverse ligament retained its hold on the two extremities of the remaining fragment of the atlas, thus protecting the spinal marrow from injury. This bone in possession of Professor Pattison I repeatedly saw and carefully examined; he exhibited it to his class, and it was mislaid or lost. At the request of Professor White, I make this statement of facts. The bone was in our possession in 1838-39-40, or thereabouts. I then understood and believed (since confirmed by conversation with Professor White) that it came from his throat, coming out through the mouth as a consequence of ulceration, the result of an accident while riding in a stagecoach on the morning of December 17th, 1837. The bone was discharged at the expiration of forty-five days after receipt of the injury."

"This statement, signed 'Joseph Pancoast,' records an interesting and remarkable accident which, so far as we know, has never been made public, and of which there are only one or two instances mentioned in literature." (*Medical News,* Nov. 27, 1886).

Comment. The last statement, quoted above from the *Medical News,* is certainly correct in that this was a most interesting and remarkable case, though just what justification there is for the further statement that there are only one or two instances on record I cannot determine. I have made a careful search through the *Index Catalogue* of the Surgeon General's Library, both series, and, unless titles are incomplete or misleading, I find there is not a single duplicate of this case reported. Bayard (*Boston M. and S. Journal,* 1870.) and Sinkler (*Philadelphia Medical Times,* 1875.) both report cases of fracture of the odontoid process with recovery, while every case of fracture of the atlas, if one may judge by the title, has sooner or later proved fatal. Through the annual volumes of the *Index Medicus* I have not taken the pains to search. At all events Dr. White's case was of the most extreme rarity, and it is most unfortunate that better details are lacking.

Of his condition during the forty-five days previous to the extrusion of the fragment there is no account, neither is there of the time elapsing before his restoration to his usual activity; but inasmuch as he died in 1881, having passed the subsequent part of his life in a most active professional career, it is legitimate to conclude that he suffered little, if at all, from the consequences of his injury.

---

Nearly Coincident Eclampsia of Mother and Daughter. M. H. Newman of Cody, Neb., *Med. Review,* Oct., 1912. Daughter, aged 20, 2-para, post-partum, July 3, urine contains trace of albumin. Mother, 7-para, aged 36, ante-partum, July 4-6, moderate albuminuria. Both recovered.

